# Incomplete Anterior Spinal Artery Syndrome Responsive to Intrathecal Baclofen

**DOI:** 10.7759/cureus.40391

**Published:** 2023-06-13

**Authors:** Andrew Waack, Maximilian Fliegner, Daniel L Menkes, Michael D Staudt

**Affiliations:** 1 Neurosurgery, The University of Toledo, Toledo, USA; 2 Neurosurgery, Oakland University William Beaumont School of Medicine, Rochester, USA; 3 Neurology, Beaumont Health, Royal Oak, USA; 4 Neurology, Oakland University William Beaumont School of Medicine, Rochester, USA; 5 Neurosurgery, Beaumont Health, Royal Oak, USA

**Keywords:** spasticity, intrathecal, endovascular repair of abdominal aneurysm, baclofen, anterior spinal artery syndrome, anterior cord syndrome, abdominal aortic stenosis

## Abstract

Anterior cord syndrome (ACS) occurs as a result of ischemia in the territory of the anterior spinal artery (ASA). Although spinal cord strokes are rare, the ASA is the most commonly affected vessel in the spinal cord. The typical presentation of an ASA stroke is paraparesis or paraplegia, bilateral loss of pain and temperature sensation, and fecal or urinary incontinence; the underlying neural structures responsible for these symptoms include the corticospinal tracts and anterior horns, anterolateral spinothalamic tracts, and lateral horns, respectively. ACS is a feared complication of aortic procedures and has been well-documented to occur during or after endovascular abdominal aortic aneurysm revascularization (EVAR). We report a case of incomplete or partial ACS presenting with delayed-onset spasticity and instability several months following EVAR, who was subsequently treated with intrathecal baclofen. We hypothesize that this patient’s ischemia selectively damaged descending white matter tracts responsible for modulating the stretch receptor reflex, including damage to the corticospinal tract, which likely also impaired positional stability.

## Introduction

The vascular supply to the spinal cord comprises a single anterior spinal artery (ASA), paired posterior spinal arteries (PSAs), and a circumferential pial arterial network from the ASA and both PSAs [1]. The rostral ASA arises from the confluence of vertebral artery branches; this extends along the length of the spinal cord until it forms an anastomotic loop with the PSAs at the conus medullaris [2,3]. The caudal ASA is variably supplied along its longitudinal axis by radiculomedullary arteries that arise from posterior intercostal, lumbar, and internal iliac arteries [2]. There is a high degree of variability in the number, location, and caliber of radiculomedullary contributions to the ASA; however, there is typically a principal radiculomedullary artery responsible for the majority of caudal ASA blood supply, known as the great anterior radiculomedullary artery, or Artery of Adamkiewicz [4]. This vessel has a characteristic “hairpin” course and usually originates from a left-sided posterior intercostal artery within the T8-L1 levels, and it is a major consideration for aortic surgery and revascularization [4,5].

Spinal cord pathology reflects the underlying topographical organization of the spinal cord. Several patterns of spinal cord ischemic injury have been described, including anterior cord syndrome (ACS), posterior cord syndrome, Brown-Sequard syndrome, and other classic spinal cord pathologies. Spinal cord ischemia is rare and comprises only 1-2% of strokes [3]. Although rare, ACS is the most common ischemic presentation. Neural structures at risk include the corticospinal and spinothalamic tracts with sparing of the dorsal columns. ACS typically presents acutely and can include paraparesis, paraplegia, bilateral loss of pain and temperature sensation, and fecal or urinary incontinence [5,6].

Many causes of ACS have been described [1,2,5,7-11]. Maintaining ASA perfusion is of particular concern during open and endovascular aortic procedures, such as endovascular abdominal aortic aneurysm repair (EVAR). Spinal cord monitoring is a well-documented mechanism for detecting intraoperative spinal cord injury. It has also been suggested that ischemic preconditioning of the spinal cord before EVAR may be protective against neurological injury [12]. ACS is a well-documented complication of these procedures [2,5,7-9]. Several techniques have been described for the management of ACS, although there are no clear guidelines. Commonly cited approaches include spinal cerebrospinal fluid drainage with a sustained elevation in mean arterial pressure, steroids, naloxone, hyperbaric oxygen, and hypothermia [5].

We describe the presentation and management of an unusual case of incomplete or partial ACS, in which the patient presented with spasticity several months following EVAR and was subsequently treated with intrathecal baclofen.

## Case presentation

We present the case of a 64-year-old man with a four-year history of bilateral claudication symptoms manifesting with leg weakness, pain, cramping, and falls. He was diagnosed with severe abdominal aortic stenosis and underwent stent grafting, which improved his symptoms for about six months. The stent extended from the T8 vertebral level down to the iliac arteries.

He reported “rubbery legs” immediately following surgery, although without significant weakness or functional limitations. Six months following endovascular treatment, he began experiencing truncal instability and spastic paraparesis which worsened with exertion. Upper extremity strength and tone were preserved. In the lower extremities, he demonstrated at least 3/5 strength proximally and 5/5 strength in toe flexors and extensors. Increased tone was observed in both lower extremities with truncal titubation that disappeared while lying flat. His gait was slightly spastic, and the Romberg sign was negative. He was able to walk on his toes but slid to the floor when attempting to walk on his heels. No lower extremity edema was observed and a full pulse examination was normal. No autonomic dysfunction was reported. The sensation was intact.

Thoracic and lumbar MRI scans were negative for pathology; specifically, no abnormal cord signal intensity was appreciated. A muscle biopsy was unremarkable. Needle electromyography (EMG) demonstrated spontaneous activity in the lower extremities and in the bilateral thoracic paraspinals, which localized the lesion to the anterior horn cells/motor nerve roots in the lower thoracic spine. The clinical finding of truncal titubation while standing but not sitting implied involvement of the spinocerebellar tracts as well.

The patient was seen by multiple specialists over the course of three years without a definitive diagnosis or clinical improvement. His treatment consisted of physical therapy and oral baclofen, which provided little relief. Given the absence of trauma or anatomic evidence of demyelination on his MRI, the etiology was presumed to be vascular in nature. Ultimately, his clinical picture was considered consistent with an incomplete or partial ASA syndrome, with ischemia of the great artery of Adamkiewicz secondary to the aortic stent placement.

A trial of intrathecal baclofen was planned. Before baclofen administration, he completed two sit-to-stand tests in 20 seconds, which required him to initiate movement with his upper extremities and momentum. Pre-procedure ambulation assessment revealed right lower extremity weakness and positive Trendelenburg sign with truncal spasticity, requiring him to reach out his right arm and turn his neck to the right to compensate for this instability (Video 1).

**Video 1:** Pre-trial ambulation assessment. Pre-trial ambulation assessment revealed right lower extremity weakness and positive Trendelenburg sign with truncal spasticity. Approximately every four to five steps, the patient’s right lower extremity would “feel weak” and trunk spasticity would increase in a rolling motion with the patient reaching his right arm out to the side.

He subsequently underwent a trial of intrathecal baclofen (50 µg), and testing began after 60 minutes. He was able to complete eight sit-to-stand tests in 30 seconds with no use of momentum, although the final two repetitions required more effort. Moreover, the patient was able to ambulate with symmetric stride length and better postural control both with and without a walker. He also reported greater confidence in coordinating his lower extremities (Videos 2, 3).

**Video 2:** Post-trial sit-to-stand test. Post-trial sit-to-stand test was markedly improved with no use of momentum and with symmetric stride length and better postural control. There were no instances of needing to steady himself or resort to reaching his arm out to the side.

**Video 3:** Post-trial sit-to-stand test without a walker. Improvements were also maintained without the use of a rolling walker. Limited arm swing is evident on the right, with slight right trunk flexion during a right stance.

The patient underwent implantation of an intrathecal programmable pump, with the catheter placed at the T8-9 level. His initial rate was set at 50 µg/day with a priming bolus programmed, and within one to two days, he reported a 90% reduction in symptoms. During the follow-up, a flex dose was added in the morning, and his total 24-hour dose was raised to 61.18 µg/day. At the 14-month follow-up, he reported a similar 90% improvement in spasticity and ataxia, characterized by improved functional mobility and increased tolerance with strength testing to proximal hip muscles with a noted decrease in trunk spasms. Of note, his overall strength has not improved, and he still experiences lower extremity weakness with exertion and toward the end of the day. Regardless, the patient reports greater confidence in mobility, particularly in regard to “coordinating the legs to move as a team.”

## Discussion

This case demonstrates that an incomplete ASA can be challenging to diagnose and may present in a delayed manner. The neurological sequelae may be improved with intrathecal baclofen therapy, particularly spasticity and postural instability.

Spinal cord ischemia in the ASA region can result in ACS. Systemic hypotension and thoracolumbar aortic or artery of Adamkiewicz pathologies, including atherosclerosis, dissection, aneurysm, and thrombosis, are common causes; iatrogenic ACS has been reported following open or endovascular aortic surgery [2,5,7-9], kyphoplasty [10], coronary angiography [11], inadvertent vessel ligation during spine surgery, and intracranial endovascular procedures [1]. Spinal cord ischemia is a feared complication of EVAR procedures; fortunately, it is a rare complication with an incidence of 0.21% [13]. Spinal cord ischemia following EVAR can occur through several mechanisms. EVAR stent deployment disrupts blood flow to the inferior mesenteric artery and infrarenal lumbar arteries, which can contribute anastomotic radiculomedullary branches to the ASA; thus, stent deployment may impair ASA perfusion [13]. Additionally, endovascular techniques can displace embolic material into the spinal cord circulation. Spinal cord ischemia is more likely to occur in patients with anatomical vascular variations that make EVAR technically challenging. Such variants often necessitate additional procedures that lengthen total operative times; performing EVAR on such patients leads to significantly worse outcomes with regard to mortality, complications, and additional interventions [13].

The pathophysiology of ASA secondary to spinal cord ischemia is complex, and various presentations have been described based on the extent of neural impairment, the timing of symptom onset, and the degree of functional recovery. ACS is a classic ischemic presentation involving the entire ASA vascular territory. Features of ACS can include paraparesis or paraplegia, bilateral loss of pain and temperature sensation, and fecal and/or urinary incontinence, reflecting disruption to the corticospinal tract and anterior horn, anterolateral spinothalamic tracts, and lateral horns, respectively [10]. The onset of symptoms following EVAR typically occurs immediately after the procedure [5]. Peak ACS symptomatology typically occurs within 12 hours of onset, and most symptoms resolve within 72 hours of onset [6]. There have been reports of delayed ACS occurring in the days, weeks, and months following EVAR [7-9]. Moulakakis et al. described 14.8% of post-EVAR ACS cases as being delayed [13]. Outcomes following ACS ranges from no improvement to total recovery; Moulakakis et al. described complete recovery in 25% of patients, minimal improvement in 50%, and no improvement in 25% of patients. Nedeltchev et al. reported 41% of patients regaining full ambulation without aids, 30% ambulation with aids, and 20% remaining wheelchair-bound at a mean of 4.5 years following acute ischemia; this group also identified severe initial impairment (classified as American Spinal Cord Injury Association A and B scores) and female gender as independent predictors of poor outcome [14].

Incomplete ACS can occur due to selective ischemic damage within the ASA territory. Isolated anterior horn ischemic injury is the most commonly described incomplete ASA, which presents as flaccid paralysis and areflexia due to lower motor neuron injury, without accompanying sensory and autonomic deficits observed in classical ACS [2,6,15]. Long-term consequences may include progressive distal amyotrophy, which presents similarly to amyotrophic lateral sclerosis [6]. Bilateral brachial diplegia (“man-in-the-barrel syndrome”) secondary to cervical anterior horn ischemia has been described secondary to vertebral artery dissection [15]. ACS presentations may vary depending on the degree of ischemic insult. MRI may aid in the diagnosis of ACS, with T2 hyperintensity visible within the region of the anterior horns. Transcranial magnetic stimulation might demonstrate an increased threshold to activation in the lower extremities and/or a prolonged motor central conduction time. In contrast, tibial somatosensory evoked potential studies would be normal, as this modality assesses the dorsal columns that are spared in an ASA syndrome. In complete ASA syndrome, needle EMG may demonstrate fibrillation potentials in all limb myotomes supplied by the ASA. In the current case, the thoracic paraspinal muscles were denervated.

Our case was particularly challenging to diagnose because of a delayed symptom onset following EVAR and the lack of classical ACS findings, particularly the delayed-onset spasticity, which is a rare manifestation following spinal ischemia [16]. Spasticity is defined by a velocity and length-dependent hyperactivity of the stretch reflex that results in increased muscle tone [17], by which muscle lengthening induces Ia afferent fiber excitation and subsequent activation of ipsilateral lower motor neurons and muscle contraction. Stretch reflex arc activity is modulated by input from descending pyramidal upper motor neurons in the corticospinal tract and parapyramidal neurons in the dorsal reticulospinal (inhibitory) and medial reticulospinal (excitatory) tracts [17]. Damage to these descending modulatory pathways interferes with the regulation of the stretch reflex, which may result in an over-excitable reflex arc and subsequent increase in muscle tone. We believe selective ischemic injury damaged the descending white matter pathways responsible for stretch reflex modulation, which resulted in an increase in stretch reflex activity and subsequent spasticity. Additionally, we believe the postural instability exhibited reflects a mismatch between afferent proprioceptive input by the dorsal columns and efferent motor output by the corticospinal tracts. Because the dorsal column function includes conscious proprioception, it is critical in maintaining postural stability. Postural instability may reflect dysfunction of either the dorsal columns or the corticospinal tract, as constant feedback between these two systems is required [18]. As this patient’s dorsal columns were spared, the observed postural instability resulted from an imbalance of proprioceptive and motor function between the dorsal columns and the corticospinal tract. The EMG data demonstrating spontaneous activity in the thoracic paraspinal muscles confirmed the loss of the anterior horn cells/motor nerve roots in this region. While the thoracic MRI was unremarkable, this degree of spinal cord ischemia is often below the resolution of 3T MRI scanners [19]. Regardless of the mechanism, this case presented a diagnostic challenge because it did not display symptoms characteristic of ACS.

Baclofen is a gamma-aminobutyric (GABA-B) receptor agonist used to treat spasticity encountered in various neurological conditions, including multiple sclerosis, spinal cord injury, and cerebral palsy [20]. GABA-B receptors can be found on the synaptic boutons of the Ia afferent axons involved in the stretch reflex arc; GABA-B activation results in axon hyperpolarization and decreased action potential frequency in the stretch reflex arc, relieving spasticity [20]. Both oral and intrathecal dosages are available. Intrathecal baclofen is often preferred for spasticity because baclofen’s poor oral bioavailability limits delivery to the spinal cord, and intrathecal delivery minimizes baclofen blood levels. We elected to use intrathecal baclofen to treat our patient’s spasticity, with a good outcome.

## Conclusions

We describe an interesting case of delayed, partial ACS presenting with spasticity which was successfully treated with intrathecal baclofen several months after EVAR. This case presented a diagnostic challenge because of its delayed onset, as it did not present with the classical signs of ACS. Nevertheless, intrathecal baclofen, a staple of spasticity management, was successfully utilized to treat the patient’s symptoms.
